# Curcumin Attenuates Testicular Injury in Rats with Streptozotocin-Induced Diabetes

**DOI:** 10.1155/2018/7468019

**Published:** 2018-07-31

**Authors:** Wenliang Zha, Yuting Bai, Ling Xu, Yuning Liu, Zhen Yang, Hui Gao, Jun Li

**Affiliations:** ^1^Department of Surgery, Clinic Medical College, Hubei University of Science and Technology, Xianning 437100, China; ^2^Hubei Province Key Laboratory on Cardiovascular, Cerebrovascular, and Metabolic Disorders, Hubei University of Science and Technology, Xianning 437100, China; ^3^National Demonstration Center for Experimental General Medicine Education, Hubei University of Science and Technology, Xianning 437100, China; ^4^School of Pharmacy, Hubei University of Science and Technology, Xianning 437100, China

## Abstract

Oxidative damage, inflammation, and apoptosis are the primary features of diabetic testicular damage. Curcumin protects against diabetic testicular injury, but the underlying mechanisms remain obscure. This study examined the effect of curcumin on type 2 diabetes mellitus- (T2DM-) induced testicular injury, oxidative stress, and apoptotic changes. T2DM rats were intraperitoneally injected with 40 mg/kg STZ after being fed a high-fat diet for 8 weeks. One week after STZ injection, 100 or 200 mg/kg curcumin was administered orally to the diabetic rats for 16 weeks. Histological changes in the testes were determined by HE staining. Serum testosterone was measured. Markers of superoxide levels, such as SOD activity and MDA content, and markers of cell death, including the expression of Bax, Bcl-2, and MAPK family members, were measured by molecular biology or immunohistochemical techniques. Degeneration and disruption of seminiferous tubule structure were observed in diabetic rats. Serum testosterone levels were markedly lower in diabetic rats than in control rats. Moreover, testicular apoptosis and Bax expression were much higher in diabetic rats than in control rats. Superoxide generation, the NADP^+^/NADPH ratio, and NADPH oxidase subunit expression, including expression of the gp91^phox^, p47^phox^, and p67^phox^ subunits, increased, while antioxidant enzyme levels decreased in diabetic rats. Furthermore, the MAPK signaling pathway was activated in diabetic rats. Curcumin partially prevented diabetes-induced microstructural abnormalities and significantly increased serum testosterone levels compared to untreated T2DM rats. Additionally, curcumin reduced testicular apoptosis by regulating apoptotic proteins and markedly inhibited oxidative stress levels by downregulating MDA expression, decreasing NADPH activity, and restoring antioxidant enzymes. Remarkably, curcumin treatment also suppressed MAPK activation. Thus, curcumin may have therapeutic value in the treatment of diabetes-induced testicular injury due to its prevention of testicular apoptosis and attenuation of oxidative stress.

## 1. Introduction

Diabetes mellitus (DM), a serious metabolic disorder, is becoming a major health problem affecting millions of people. In fact, the global incidence of DM is projected to reach 642 million by 2040 [1]. Although many researchers mainly pay attention to the most common complications of diabetes, such as diabetic cardiomyopathy, nephropathy, and retinopathy, researchers are becoming increasingly aware of male sexual and reproductive dysfunction, which occurs in both diabetic animals and patients [2, 3]. Low testosterone levels and testicular damage can lead to erectile dysfunction, decreased sperm motility, and reduced seminal fluid volume in the context of DM [4]. Even though diabetes-induced testicular dysfunction is one of the most prevalent diabetic complications, the underlying mechanisms in the testes that lead to hypogonadism have not been thoroughly elucidated. Apoptosis or programmed cell death is a form of cell death that eliminates dying cells from the proliferating or differentiating cell population; this process is thought to play a pivotal role in the pathogenesis of hypogonadism [5]. Apoptotic cell death is significantly increased in the seminiferous tubules of streptozotocin- (STZ-) induced diabetic mice and rats and is considered one of the major reasons underlying infertility in diabetic animals [6, 7].

Oxidative stress contributes to the pathophysiology of impaired spermatogenesis and loss of germ cells [8, 9]. Both reactive oxygen species (ROS) overproduction and diminished antioxidant defenses may result from excessive oxidative stress in diabetic testes. If the balance between ROS generation and ROS scavenging systems is lost, superoxide accumulation will eventually cause cellular damage or dysfunction. Additionally, diabetes-mediated oxidative stress can induce apoptosis [10]. Given the adverse effects of oxidative stress, there is growing interest in the use of antioxidants as potentially beneficial therapeutic agents [11].

Curcumin, a yellow curry pigment isolated from the root of Curcuma longa has well-known powerful antioxidant properties. In recent years, curcumin has attracted attention because it possesses a variety of beneficial biological properties, such as anti-inflammatory [12], antitumor [13], and antidiabetic [14] activities. Evidence supports the benefits of 100 mg/kg curcumin against testicular injury from STZ-induced diabetes via the inhibition of oxidative stress [15–17]. However, whether curcumin plays a beneficial role in testicular injury by reducing nicotinamide adenine dinucleotide phosphate (NADPH) oxidase remains unknown. Therefore, this study was designed to evaluate the effect of different doses of curcumin on STZ-induced diabetic testicular injury, oxidative stress, apoptosis, and changes in NADPH oxidase.

## 2. Materials and Methods

### 2.1. Experimental Animals

Male Wistar rats, at approximately 4-5 weeks of age and weighing 70-90 g, were provided by the Laboratory Animal Center of the Preventive Medicine Academy of Hubei Province. This study was conducted in accordance with the National Institutes of Health guidelines. All animal procedures used here were approved by the Institutional Animal Care and Use Committee of Hubei University of Science and Technology.

### 2.2. Type 2 DM Induction and Treatment

Rats were randomly divided into the following groups: nondiabetic group (control, n=10), diabetes group (DM, n=20), low-dose curcumin treatment group (DM+Cur 100 mg/kg group, n=12), and high-dose curcumin treatment group (DM+Cur 200 mg/kg group, n=12). Control rats were fed a basal diet for 8 weeks and then received injections of vehicle (0.1 mol/L citrate buffer). DM rats were fed a high-fat diet for 8 weeks and then intraperitoneally injected with 40 mg/kg STZ (dissolved in 0.1 mol/L citrate buffer, pH 4.5). Rats with blood glucose levels≥11.6 mmol/L were considered diabetic. DM+Cur groups were intragastrically given 100 or 200 mg/kg/day curcumin mixed with 0.5% sodium carboxymethyl cellulose (CMC-Na) to form a suspension for 16 weeks after one week of STZ injection. The DM-only and control groups were intragastrically given vehicle (CMC-Na) only. All animals were fed a basal diet from the time of STZ injection until the completion of the experiment.

After the experiments, the rats were anesthetized with 5 mg/kg urethane and euthanized. Blood samples were collected, and the serum was separated. Testes were rapidly removed for histopathological detection. The remaining tissue was stored at −80°C for western blot analysis.

### 2.3. Measurement of Testosterone Level

Serum testosterone hormone levels were determined using a testosterone enzyme-linked immunosorbent assay (ELISA) kit (CUSABIO Biotechnology Co. Ltd, China) in accordance with the manufacturer's instructions.

### 2.4. Histopathological Examination

Testes were fixed in Bouin's solution overnight at 4°C, embedded in paraffin, sectioned into 5-*μ*m thick slices, deparaffinized, and stained with hematoxylin and eosin (HE). The morphology of the testes was evaluated under a light microscope.

### 2.5. Determination of Biochemical Indices

Testis tissue was harvested and homogenized in 50 mmol/L phosphate buffer. Subsequently, the homogenate was centrifuged at 3000× g at 4°C for 15 min. The levels of superoxide dismutase (SOD), malondialdehyde (MDA), and glutathione peroxidase (GSH-Px) in the resultant supernatant were measured according to the appropriate detection kits purchased from Nanjing Jiancheng Bioengineering Institute (China) [18].

### 2.6. Terminal Deoxynucleotidyl Transferase-Mediated dUTP Nick End-Labeling (TUNEL) Assay

TUNEL assays were performed following the manufacturer's instructions (Roche Applied Science, USA). Briefly, samples were dewaxed with xylene, rehydrated using 100% to 50% ethanol, incubated in 1% Triton X-100 at room temperature for 15 min, and then placed in 3% H_2_O_2_-methanol solution at room temperature for 10 min. Subsequently, 20 mg·mL^−1^ proteinase K was added to sections and incubated for 15 min. The sections were then incubated with TdT-enzyme at 37°C for 1 h and washed with phosphate-buffered saline (PBS) three times. Then, 100 *μ*L of digoxigenin (conjugated to horseradish peroxidase, POD) was placed on each section. 3, 3′-Diaminobenzidine (DAB) was used as a staining agent. Apoptotic cells were stained brown and analyzed by randomly counting TUNEL-positive cells under a fluorescence microscope at 400× magnification (Olympus BX53, Japan) [19].

### 2.7. Immunohistochemical Determination of Bcl-2 and Bax Expression

Samples were sliced into 5-*μ*m thick sections and mounted onto glass slides. B cell lymphoma 2 (Bcl-2) and Bcl-2-associated X (Bax) primary antibodies (1:200, Santa Cruz Biotechnology, USA) were applied to each sample, and the samples were incubated at 4°C overnight. After three washes with PBS, a biotinylated horse anti-mouse immunoglobulin G (IgG) solution was added to the samples and incubated for 30 min. The samples were then incubated with avidin-biotin-peroxidase complexes. The reaction was visualized after incubation with a DAB solution. Images were captured under a microscope at 200× magnification.

### 2.8. Measurement of Superoxide in Testes by DHE Staining

*Testicular tissue* was embedded in an optimal cutting temperature gel, sliced to a thickness of 5 *μ*m with a cryotome, and then placed on glass slides. Specimens were incubated in a light-impermeable chamber at 37°C for 30 min after application of 10***μ***M dihydroethidium (DHE, Life Technology, USA) and washed twice with PBS. In the presence of superoxide, DHE is converted to the red fluorescent 2-hydroxyethidium molecule. Images of testicular tissue (400× magnification) were captured using an inverted fluorescence microscope (Olympus IX71, Japan).

### 2.9. Determination of the NADP^+^/NADPH Ratio

Because NADPH oxidase activation is closely related to the promotion of oxidant production, we evaluated NADPH oxidase activity and the expression of some of its isoforms. NADP and NADPH levels were determined using an EnzyChromTM NADP^+^/NADPH assay kit (Bioassay Systems, CA) according to the manufacturer's instructions. The assay is based on a glucose dehydrogenase cycling reaction. Concentrations of NADP^+^ and NADPH were measured at 565 nm and recorded with a Bio-tek ELx800.

### 2.10. Western Blot Analysis

Testicular tissue was homogenized in 1× radioimmunoprecipitation assay (RIPA) lysis buffer (Cell Signaling Technology, USA) and centrifuged at 12000 rpm for 15 min at 4°C. Protein concentrations in the supernatant were measured by a bicinchoninic (BCA) protein assay (Beyotime, China). An equal concentration of protein was loaded and separated by sodium dodecyl sulfate-polyacrylamide gel electrophoresis (SDS-PAGE), and the following antibodies were used for western blotting: Bcl-2, Bax, p67^phox^, p38 mitogen-activated protein kinase (MAPK), and phospho-p38 MAPK (Thr180/Tyr182); Jun NH2-terminal kinase (JNK) and phosphor-JNK (Thr183/Tyr185); extracellular signal-related kinase (Erk)1/2MAPK and phosphor-Erk1/2 MAPK (Thr202/Tyr204) (1:1000, Cell Signaling Technology, USA); and p47^phox^ and gp91^phox^ (1:200, Santa Cruz Biotechnology, USA). The membranes were then incubated with the appropriate secondary antibodies for 1 h at 37°C. Blots were visualized with enhanced chemiluminescence (ECL) kits (Pierce Biosciences, USA).

### 2.11. Statistical Analysis

Data are presented as the mean±SD from repeated experiments. Statistical analysis was performed using one-way analysis of variance (ANOVA). Differences were considered statistically significant at a* P* value<0.05.

## 3. Results

### 3.1. Curcumin Improved the Basic Conditions of DM Rats

After the 16-week treatment period, eight rats died in the DM group, while three and two rats died in the 100 mg/kg and 200 mg/kg curcumin treatment groups, respectively. These rats died due to diabetic ketoacidosis, infection, or cardiomyopathy. Throughout the experimental period, control group rats had white or light-colored fur. In the DM group, the fur of the rats gradually lost its luster and became dull. Moreover, three rats had diabetic complications such as cataracts. Compared with rats in the DM group, rats in the DM+Cur groups exhibited shiny fur and improvements in all symptoms.

### 3.2. Curcumin Prevented Metabolic Changes in Diabetic Rats

**Figure 1:**
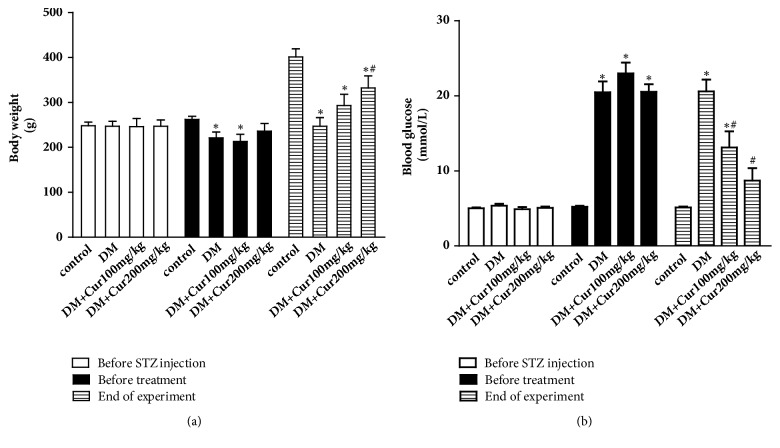
**Curcumin prevented metabolic changes in diabetic rats**. (a) Curcumin elevated the body weight of rats. (b) Curcumin reduced the blood glucose levels in rats. n=7-9. Values are presented as the mean±SD. ^*∗*^*P*<0.05 versus the control group. ^#^*P*<0.05 versus the DM group.

The DM group exhibited typical symptoms of diabetes, including hyperglycemia, polydipsia, and polyuria. As shown in Figure 1, body weight was remarkably reduced, while the level of blood glucose was significantly increased in the DM group (*P*<0.05 versus the control group). These metabolic abnormalities were reversed in all of the curcumin treatment groups in a dose-dependent manner (*P*<0.05 versus the DM group).

### 3.3. Curcumin Ameliorated the DM-Induced Reduction of Testosterone Hormone

The serum testosterone levels of different groups are shown in Table 1. Compared with the control group, the DM group showed a significant decrease in the serum testosterone concentration (*P*<0.05). In addition, the serum testosterone concentration was significantly higher (*P*<0.05) in the curcumin treatment groups than in the DM group.

### 3.4. Curcumin Attenuated DM-Induced Pathological Changes

**Figure 2:**
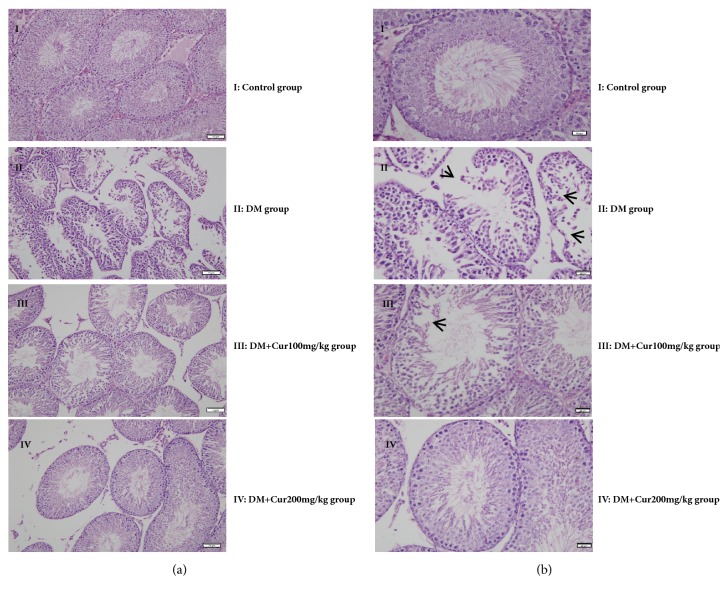
**Histological changes in testes visualized using hematoxylin-eosin staining**. (a) Representative images at 200× magnification; bar indicates 50 *μ*m. n=5. (b) Representative images at 400× magnification; bar indicates 20 *μ*m. n=5. (I) Control group; (II) STZ-diabetes group; (III) treated with a low dose of curcumin; and (IV) treated with a high dose of curcumin (magnification=400×).

Histological sections from the testes of all groups are presented in Figure 2. Examination of HE-stained sections of testicular tissue from the control group showed normal testicular structure. Germinal cells in the testes of diabetic rats showed abnormal changes, including degeneration, disorganization, and interstitial edema. Low-dose curcumin treatment ameliorated the testicular histological damage, with the exception of germinal disorganization, interstitial edema, and congestion. High-dose curcumin treatment resulted in normal histology and architecture.

### 3.5. Curcumin Decreased DM-Induced Apoptosis in the Testis

**Figure 3:**
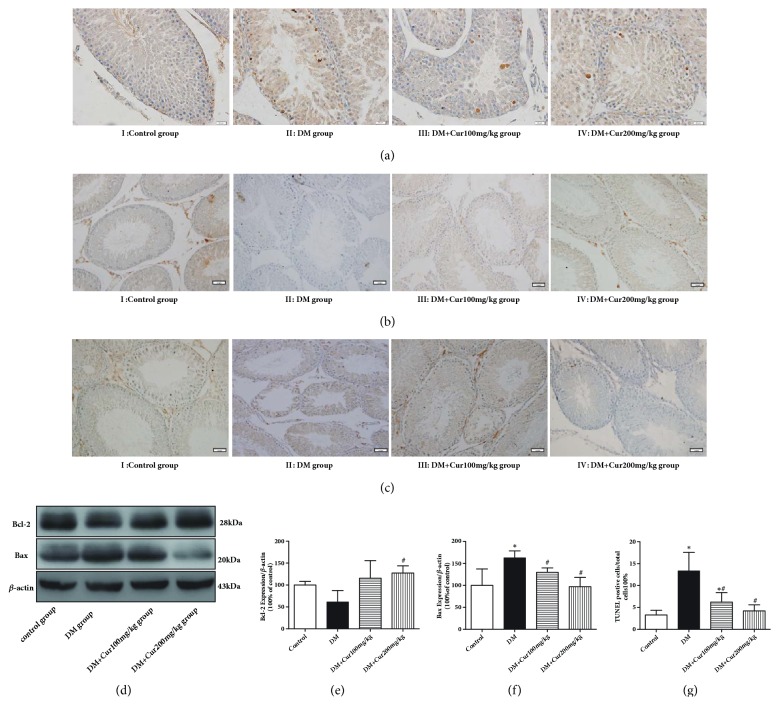
**Curcumin decreased DM-induced apoptosis in the testis**. (a) Representative images of apoptotic cells stained by TUNEL (magnification=400×; bar indicates 20 *μ*m). (b) Representative immunohistochemical staining of Bcl-2 (magnification=200×; bar indicates 50 *μ*m). (c) Representative immunohistochemical staining of Bax (magnification=200×; bar indicates 50 *μ*m). (d) Representative images of Bcl-2 and Bax protein; *β*-actin served as the loading control. (e) Quantitative analysis of Bcl-2 expression. (f) Quantitative analysis of Bax expression. (g) Analysis of TUNEL-positive cells. n=4. Values are presented as the mean±SD. ^*∗*^*P*<0.05 versus the control group. ^#^*P*<0.05 versus the DM group.

Apoptosis in the different groups, as indicated by TUNEL-positive staining, is presented in Figure 3(a). In the present study, TUNEL-positive cells were rarely observed in the control group. Germ cells demonstrated the typical morphological features of apoptosis, including chromatin condensation and cytoplasmic budding; these cells were stained brown and were markedly observable in the testes of DM rats. Curcumin administration remarkably decreased the number of apoptotic germ cells, confirming its protective effect by targeting apoptosis.

To further assess the impact of curcumin on testis survival, we analyzed apoptosis-related proteins by immunohistochemistry and western blot analysis. As shown in Figures 3(b)–3(f), Bcl-2 was downregulated and Bax was upregulated in the DM group. Compared with the DM group, the DM+Cur groups exhibited a significant decrease in Bax and an increase in Bcl-2.

### 3.6. Curcumin Inhibited DM-Induced Oxidative Stress

**Figure 4:**
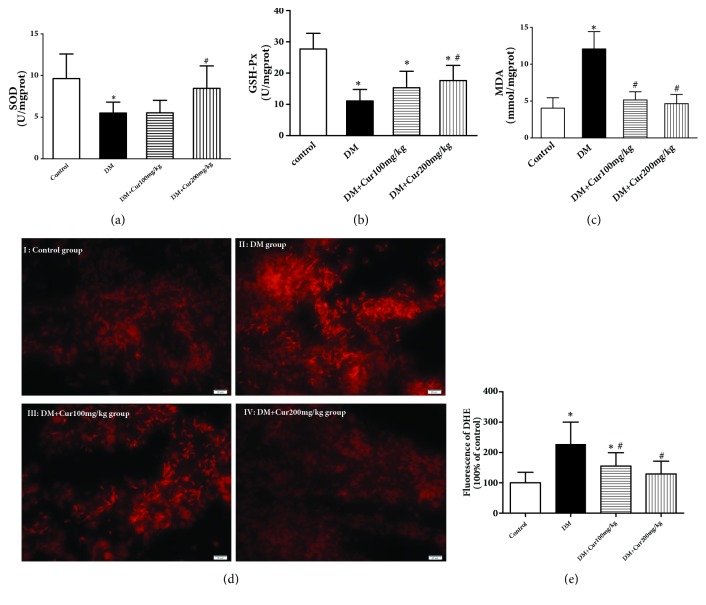
**Curcumin inhibited DM-induced oxidative stress**. (a) Curcumin elevated SOD activity in testes. (b) Curcumin enhanced GSH-Px activity in testes. (c) Curcumin reduced the MDA content in testes. (d) Representative images of DHE staining. (e) Analysis of the fluorescence signal from DHE, n=4. Values are presented as the mean±SD. ^*∗*^*P*<0.05 versus the control group. ^#^*P*<0.05 versus the DM group.

As shown in Figures 4(a)–4(c), there was greater accumulation of lipid peroxides in the testes of rats in the DM group, with a concordant reduction in both SOD and GSH-Px activities as well as an increase in MDA release(*P*<0.05 versus the control group). After treatment with curcumin, both SOD and GSH-Px activities were significantly elevated, and the concentration of MDA was greatly reduced. The observed difference was statistically significant (*P*<0.05 versus the DM group).

ROS, a key contributor to oxidative stress, was detected with DHE staining. As presented in Figure 4(d), superoxide fluorescence, which is representative of the ROS concentration, was significantly increased in the testes of the DM group. After treatment with curcumin, a decrease in superoxide fluorescence was observed.

### 3.7. Curcumin Attenuated DM-Induced NADPH Oxidase Isoform Expression and Decreased the NADP^+^/NADPH Ratio in Testes

**Figure 5:**
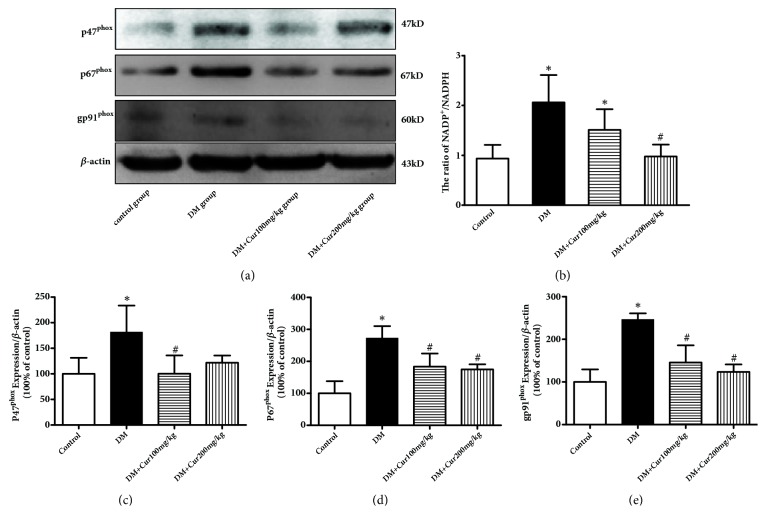
**Curcumin inhibited NADPH oxidase activity and subunit expression**. (a) Representative images of p47^phox^, p67^phox^, and gp91^phox^ protein. (b) Curcumin reduced the NADP^+^/NADPH ratio. (c) Quantitative analysis of p47^phox^ expression. (d) Quantitative analysis of p67^phox^ expression. (e) Quantitative analysis of gp91^phox^ expression. n=4-5 per group. Values are presented as the mean±SD. ^*∗*^*P*<0.05 versus the control group. ^#^*P*<0.05 versus the DM group.

As shown in Figure 5, the ratio of NADP^+^/NADPH and the expression levels of p47^phox^, p67^phox^, and gp91^phox^ were significantly increased in the DM group compared with those in the control group. The ratio of NADP^+^/NADPH and the expression levels of p47^phox^, p67^phox^, and gp91^phox^ were markedly inhibited in the curcumin administration groups compared with those in the DM group (*P*<0.05). From these results, we concluded that the protective effect of curcumin against DM-induced testis injury is largely attributed to the suppression of NADPH oxidase-mediated ROS production.

### 3.8. Curcumin Inhibited DM-Induced Mitogen-Activated Protein Kinase (MAPK) Pathway Activation in Testes

**Figure 6:**
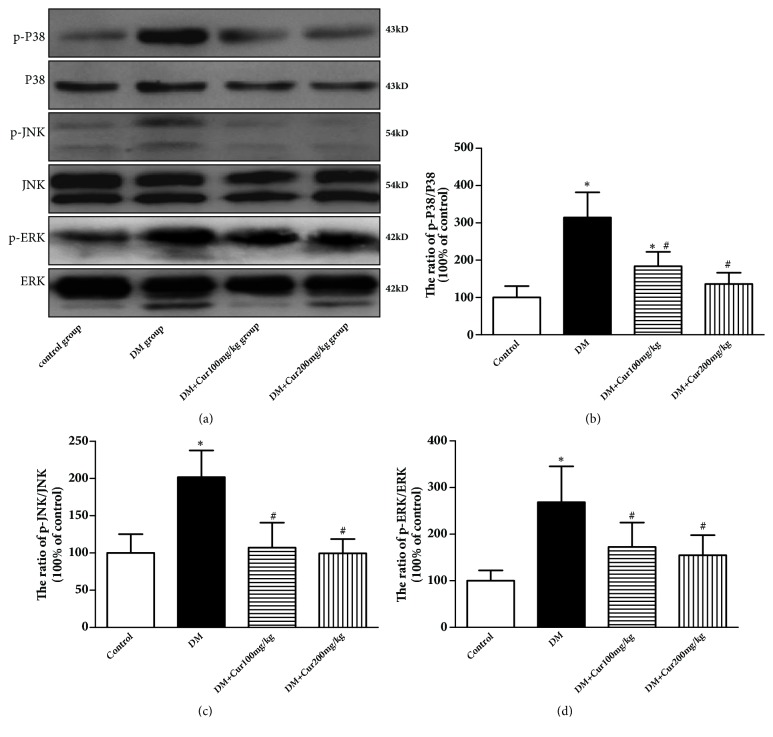
**Curcumin inhibited the MAPK pathway**. (a) Representative images of p-p38, p38, p- JNK, JNK, p-ERK, and ERK. (b) Quantitative analysis of the p-38/p38 ratio. (c) Quantitative analysis of the p-JNK/JNK ratio. (d) Quantitative analysis of the p-ERK/ERK ratio. n=3-4 per group. Values are presented as the mean±SD. ^*∗*^*P*<0.05 versus the control group. ^#^*P*<0.05 versus the DM group.

The MAPK pathway is involved in both cell survival and apoptosis. To determine the protective effects of curcumin, we measured the expression of MAPK family members, including JNK, p38MAPK, and ERK, in the different groups. As shown in Figure 6, a greater increase in the phosphorylated forms of p38, JNK, and ERK1/2 was observed in the DM group than those of control group (*P*<0.05). However, curcumin administration significantly alleviated the diabetes-induced activation of MAPK in the testes (*P*<0.05).

## 4. Discussion

The salient findings from our study suggested that curcumin exerts a protective effect on STZ-induced testicular impairment by increasing testosterone hormone secretion, ROS/O2^−^ production, and apoptosis. Glucose toxicity-induced testicular injury is believed to contribute to oxidative stress in diabetes. However, treatment of testicular injury remains challenging; curcumin may serve as an alternative treatment for testicular injury in diabetes. In a previous study, researchers used 100 mg/kg curcumin to treat diabetic rats and found that curcumin inhibited lipid peroxidation; however, the mechanism underlying the beneficial effect of curcumin on ROS in the testis was not elucidated [15–17]. In our study, we first used different doses of curcumin to determine its effect on STZ-induced testicular injury and found that 200 mg/kg curcumin induced greater improvement than 100 mg/kg curcumin, indicating a dose-dependent antioxidant effect from curcumin as well as inhibition of ROS generation. Our data also revealed that inhibition of NADPH oxidase in the testes is an advantageous effect of curcumin.

Accumulating evidence has demonstrated that diabetes plays an essential role in the etiology of testicular dysfunction by causing atrophy of seminiferous tubules and loss of spermatogenetic cells [20], which serve as morphological markers of spermatogenesis malfunction [21]. In our study, serum testosterone levels were decreased, and noticeable morphological changes in testes, including disruption of the structure of seminiferous tubules and a considerable reduction in the number of spermatogenic cells, were observed in diabetic rats. Curcumin treatment attenuated testicular injury by increasing serum testosterone levels and mitigating these pathologic alterations.

Our data suggest that diabetes induces apoptosis in testicular tissue in a manner somewhat similar to that in earlier reports [6, 22]. Apoptosis is considered the main contributing factor to the pathogenesis of testicular injury in experimental animals with DM. Thus, inhibition of diabetes-induced apoptosis is believed to represent a potential therapeutic approach for the treatment of testicular damage. Bcl-2, an antiapoptotic protein, inhibits mitochondrial apoptosis by suppressing Bax oligomerization followed by inhibition of cytochrome c release. Diabetes increases apoptotic cell death in testicular tissue through upregulation or downregulation of Bcl-2 family proteins [23]. Zhao et al. also supported this finding by reporting a marked increase in the Bax/Bcl-2 ratio in diabetic testes [24]. In parallel with morphological changes, curcumin reduces the number of TUNEL-positive cells by increasing Bcl-2 expression and decreasing Bax expression in the testes of diabetic rats. Thus, curcumin potentially exerts protective effects through inhibition of mitochondria-dependent apoptosis.

Cumulative evidence suggests that diabetes-induced testicular apoptosis is mostly due to elevated oxidative stress [25]. Oxidative stress induces testicular damage in many ways. Increased ROS production induces lipid peroxidation and mitochondrial lesions in germ cells, leading to dysfunction in testicular spermatogenesis and steroidogenesis [26, 27]. Moreover, increased ROS generation leads to DNA damage and germ cell abnormalities due to its genotoxic effects in the testes [28]. Therefore, ameliorating oxidative stress in diabetic rats represents a method to attenuate testicular injury. In the present study, endogenous antioxidant enzymes, including SOD and GSH-Px, were suppressed, while the lipid peroxidation biomarker, MDA, was significantly elevated in the testes of diabetic rats. Moreover, the present data clearly confirmed the marked increase in ROS in diabetic rat testes. These results are consistent with the increased cellular oxidative stress and lipid peroxide accumulation previously demonstrated in experimental diabetic animals [28].

NADPH oxidase is composed of four cytosolic subunits (p40^phox^, p47^phox^, p67^phox^, and Rac1) and two membrane subunits (gp91^phox^ and p22^phox^). Active oxidase (O^2-^) generates superoxide by transferring reducing equivalents from NADPH or NADH to oxygen [29]. A body of evidence has indicated that NADPH oxidase is broadly expressed in tissues and is the major source of ROS in diabetic animals [30, 31]. ROS derived from NADPH oxidase plays a key role in boosting the development of diabetes-induced testicular damage. Xu M et al. demonstrated that the level of NADPH oxidase subunits p22^phox^, p47^phox^, and p67^phox^ was significantly higher in diabetic testes than in normal testes and that elevated NADPH oxidase activity leads to significant diabetes-induced testicular damage [32]. Consistent with that report, our study showed that the expression of NADPH oxidase isoforms p47^phox^, p67^phox^, and gp91^phox^ was higher and that the NADP^+^/NADPH ratio was also significantly higher in diabetic testes. Moreover, curcumin effectively reversed the diabetes-induced overactivation of NADPH oxidase, decreasing the expression of isoforms p47^phox^, p67^phox^, and gp91^phox^. Thus, sustained NADPH oxidase inactivation during curcumin treatment could potentially contribute to the suppression of ROS generation.

MAPK acts as a pivotal regulator of cell apoptosis- and cell proliferation-related signaling pathways. P38 MAPK activation is involved in diabetes-induced testicular apoptotic cell death through the mitochondrial cell death pathway [24]. JNK is activated by cellular stress conditions, such as oxidative stress, and participates in mitochondrial apoptosis by increasing the translocation of Bax to the mitochondria, thereby promoting mitochondrial pore formation in diabetic animals [33]. Recent reports have demonstrated that increased phosphorylation of ERK is closely associated with testicular damage in streptozotocin-induced diabetic rats [34, 35]. Therefore, we tested the levels of these proteins to investigate their role in diabetes-induced testicular dysfunction. MAPK was also activated in the testes of diabetic animals, and curcumin inhibited the diabetes-induced activation of MAPK. Curcumin may help suppress the MAPK pathway and ultimately block apoptosis to exert its protective effect.

In conclusion, our study provided convincing evidence that curcumin rescues STZ-induced testicular damage, oxidative stress, and apoptosis possibly via regulation of NADPH oxidase activity and the MAPK pathway. These outcomes demonstrate that curcumin is a potential therapeutic agent for the recovery of STZ-induced testicular injury. Thus, these findings merit further study in a more clinically relevant setting.

## Figures and Tables

**Table 1:** Serum testosterone concentration in different groups.

**Groups**	**Testosterone (ng/ml)**
Control	7.24 ± 1.09
DM	1.23 ± 0.36^*∗*^
DM+Cur 100 mg/kg	2.88 ± 0.82^*∗*#^
DM+Cur 200 mg/kg	4.70 ± 1.24^*∗*#^

Control: control group; DM: diabetes model group; DM+Cur 100 mg/kg: low-dose curcumin treatment group (100 mg/kg); and DM+Cur 200 mg/kg: high-dose curcumin treatment group (200 mg/kg). Values are presented as the mean±SD. ^*∗*^*P*<0.05 versusthe control group. ^#^*P*<0.05 versusthe DM group. n=7-9.

## Data Availability

The data used to support the findings of this study are available from the corresponding author upon request.

## References

[1] Ogurtsova K., da Rocha Fernandes J., Huang Y. (2017). IDF Diabetes Atlas: Global estimates for the prevalence of diabetes for 2015 and 2040. *Diabetes Research and Clinical Practice*.

[2] Agbaje I. M., Rogers D. A., McVicar C. M. (2007). Insulin dependant diabetes mellitus: implications for male reproductive function. *Human Reproduction*.

[3] Kanter M., Aktas C., Erboga M. (2012). Protective effects of quercetin against apoptosis and oxidative stress in streptozotocin-induced diabetic rat testis. *Food and Chemical Toxicology*.

[4] Feyli S. A., Ghanbari A., Keshtmand Z. (2017). Therapeutic effect of pentoxifylline on reproductive parameters in diabetic male mice. *Andrologia*.

[5] Hikim A. P. S., Swerdloff R. S. (1999). Hormonal and genetic control of germ cell apoptosis in the testis. *Reviews of Reproduction*.

[6] Guneli E., Tugyan K., Ozturk H., Gumustekin M., Cilaker S., Uysal N. (2008). Effect of melatonin on testicular damage in streptozotocin-induced diabetes rats. *European Surgical Research*.

[7] Sainio-Pöllänen S., Henriksén K., Parvinen M., Simell O., Pöllänen P. (1997). Stage-specific degeneration of germ cells in the seminiferous tubules of non-obese diabetic mice. *International Journal of Andrology*.

[8] Cai L., Chen S. L., Evans T., Deng D. X., Mukherjee K., Chakrabarti S. (2000). Apoptotic germ-cell death and testicular damage in experimental diabetes: prevention by endothelin antagonism. *Urolithiasis*.

[9] Tsounapi P., Saito M., Dimitriadis F. (2012). Antioxidant treatment with edaravone or taurine ameliorates diabetes-induced testicular dysfunction in the rat. *Molecular and Cellular Biochemistry*.

[10] Kilarkaje N., Al-Bader M. M. (2014). Diabetes-induced oxidative DNA damage alters p53-p21^CIP1/Waf1^ signaling in the rat testis. *Reproductive Sciences*.

[11] Heeba G. H., Hamza A. A. (2015). Rosuvastatin ameliorates diabetes-induced reproductive damage via suppression of oxidative stress, inflammatory and apoptotic pathways in male rats. *Life Sciences*.

[12] Ramsewak R. S., DeWitt D. L., Nair M. G. (2000). Cytotoxicity, antioxidant and anti-inflammatory activities of curcumins I-III from Curcuma longa. *Phytomedicine*.

[13] Tian B., Wang Z., Zhao Y. (2008). Effects of curcumin on bladder cancer cells and development of urothelial tumors in a rat bladder carcinogenesis model. *Cancer Letters*.

[14] Asai A., Miyazawa T. (2001). Dietary curcuminoids prevent high-fat diet-induced lipid accumulation in rat liver and epididymal adipose tissue. *Journal of Nutrition*.

[15] Kanter M., Aktas C., Erboga M. (2013). Curcumin attenuates testicular damage, apoptotic germ cell death, and oxidative stress in streptozotocin-induced diabetic rats. *Molecular Nutrition & Food Research*.

[16] Zhao L., Gu Q., Xiang L. (2017). Curcumin inhibits apoptosis by modulating Bax/ Bcl-2 expression and alleviates oxidative stress in testes of streptozotocin-induced diabetic rats. *Therapeutics and Clinical Risk Management*.

[17] Rashid K., Sil P. C. (2015). Curcumin ameliorates testicular damage in diabetic rats by suppressing cellular stress-mediated mitochondria and endoplasmic reticulum-dependent apoptotic death. *Biochimica et Biophysica Acta (BBA) - Molecular Basis of Disease*.

[18] Yu W., Wu J., Cai F. (2012). Curcumin alleviates diabetic cardiomyopathy in experimental diabetic rats. *PLoS ONE*.

[19] Long L., Qiu H., Cai B. (2018). Hyperglycemia induced testicular damage in type 2 diabetes mellitus rats exhibiting microcirculation impairments associated with vascular endothelial growth factor decreased via PI3K/Akt pathway. *Oncotarget *.

[20] Cameron D. F., Murray F. T., Drylie D. D. (1985). Interstitial compartment pathology and spermatogenic disruption in testes from impotent diabetic men. *The Anatomical Record*.

[21] Sisman A. R., Kiray M., Camsari U. M. (2014). Potential Novel Biomarkers for Diabetic Testicular Damage in Streptozotocin-Induced Diabetic Rats: Nerve Growth Factor Beta and Vascular Endothelial Growth Factor. *Disease Markers*.

[22] Jiang X., Bai Y., Zhang Z., Xin Y., Cai L. (2014). Protection by sulforaphane from type 1 diabetes-induced testicular apoptosis is associated with the up-regulation of Nrf2 expression and function. *Toxicology and Applied Pharmacology*.

[23] Koh P.-O. (2007). Streptozotocin-induced diabetes increases the interaction of Bad/Bcl-XL and decreases the binding of pBad/14-3-3 in rat testis. *Life Sciences*.

[24] Zhao Y. G., Tan Y., Dai J. Y. (2011). Exacerbation of diabetes-induced testicular apoptosis by zinc deficiency is most likely associated with oxidative stress, p38 MAPK activation, and p53 activation in mice. *Toxicology Letters*.

[25] Wang Y., Zhang Z., Guo W. (2014). Sulforaphane reduction of testicular apoptotic cell death in diabetic mice is associated with the upregulation of Nrf2 expression and function. *American Journal of Physiology-Endocrinology and Metabolism*.

[26] Aitken R. J., Harkiss D., Buckingham D. (1993). Relationship between iron-catalysed lipid peroxidation potential and human sperm function. *Journal of Reproduction and Fertility*.

[27] Diemer T., Allen J. A., Hales H. K., Hales D. B. (2003). Reactive oxygen disrupts mitochondria in MA-10 tumor leydig cells and inhibits steroidogenic acute regulatory (STAR) protein and steroidogenesis. *Endocrinology*.

[28] Rajesh Kumar T., Doreswamy K., Shrilatha B., Muralidhara (2001). Oxidative stress associated DNA damage in testis of mice: Induction of abnormal sperms and effects on fertility. *Mutation Research - Genetic Toxicology and Environmental Mutagenesis*.

[29] Amaral S., Oliveira P. J., Ramalho-Santos J. (2008). Diabetes and the impairment of reproductive function: Possible role of mitochondria and reactive oxygen species. *Current Diabetes Reviews*.

[30] Li J., Zhu H., Shen E., Wan L., Arnold J. M. O., Peng T. (2010). Deficiency of Rac1 blocks NADPH oxidase activation, inhibits endoplasmic reticulum stress, and reduces myocardial remodeling in a mouse model of type 1 diabetes. *Diabetes*.

[31] Gray S. P., di Marco E., Okabe J. (2013). NADPH Oxidase 1 plays a key role in diabetes mellitus-accelerated atherosclerosis. *Circulation*.

[32] Xu M., Dai D. Z., Zhang Q., Cheng Y. S., Dai Y. (2010). Upregulated NADPH oxidase contributes to diabetic testicular complication and is relieved by strontium fructose 1,6-diphosphate. *Experimental and Clinical Endocrinology & Diabetes*.

[33] Faid I., Al-Hussaini H., Kilarkaje N. (2015). Resveratrol alleviates diabetes-induced testicular dysfunction by inhibiting oxidative stress and c-Jun N-terminal kinase signaling in rats. *Toxicology and Applied Pharmacology*.

[34] Kong W.-Y., Tong L.-Q., Zhang H.-J. (2016). The calcium-sensing receptor participates in testicular damage in streptozotocin-induced diabetic rats. *Asian Journal of Andrology*.

[35] Maremanda K. P., Khan S., Jena G. B. (2016). Role of zinc supplementation in testicular and epididymal damages in diabetic rat: involvement of Nrf2, SOD1, and GPX5. *Biological Trace Element Research*.

